# Complete Genome Sequences of the *p1* Gene Type 2b and 2c Strains *Mycoplasma pneumoniae* KCH-402 and KCH-405

**DOI:** 10.1128/genomeA.00513-17

**Published:** 2017-06-15

**Authors:** Tsuyoshi Kenri, Masato Suzuki, Atsuko Horino, Tsuyoshi Sekizuka, Makoto Kuroda, Hiroyuki Fujii, Toru Hashimoto, Hiroshi Nakajima, Hitomi Ohya, Keigo Shibayama

**Affiliations:** a Department of Bacteriology II, National Institute of Infectious Diseases, Tokyo, Japan; b Antimicrobial Resistance Research Center, National Institute of Infectious Diseases, Tokyo, Japan; c Pathogen Genomics Center, National Institute of Infectious Diseases, Tokyo, Japan; d Kurashiki Central Hospital, Okayama, Japan; e Okayama Prefectural Institute for Environmental Science and Public Health, Okayama, Japan; f Kanagawa Prefectural Institute of Public Health, Kanagawa, Japan

## Abstract

Here, we present the complete genome sequences of *Mycoplasma pneumoniae* KCH-402 and KCH-405, which are *p1* gene type 2b and 2c strains, respectively. These strains harbor variations in the *orf6* gene, which encodes the cytadherence-related proteins P40 and P90.

## GENOME ANNOUNCEMENT

*Mycoplasma pneumoniae*, a common cause of pneumonia and bronchitis in humans (1–3), is classified into several types according to the sequence polymorphism of the *p1* gene, which encodes a major adhesin P1 protein. KCH-402 and KCH-405, two *M. pneumoniae* strains isolated in Japan, are *p1* gene type 2b and 2c strains, respectively. The *p1* gene type 2b sequence, originally detected in Germany (4, 5), is also referred to as type 2v (6). The sequence of *p1* gene type 2c was identified in Dutch and Chinese strains (7, 8). Currently, type 2c is among the most prevalent clinical isolates in Japan; type 2b strains are rare.

We extracted genomic DNA from KCH-402 and KCH-405 cultured in PPLO medium (Becton Dickinson, Sparks, MD, USA) using the QIAamp DNA mini kit (Qiagen, Hilden, Germany). DNA libraries with insert sizes of 300 to 500 bp were prepared using the Nextera XT sample preparation kit (Illumina, San Diego, CA, USA). Genome sequencing was performed using the Illumina HiSeq 4000 platform. Paired-end reads (2 × 150 bp) were assembled *de novo* using CLC Genomics Workbench version 8.0.2 (Qiagen). Assembled contigs were mapped on the type 2a reference genome of strain 309 (GenBank accession no. AP012303.1) to identify gap regions. PCR amplicons containing gap regions were amplified and sequenced by Sanger sequencing and used to assemble the complete genome sequences. The complete genomes of KCH-402 and KCH-405 were 817,074 and 817,099 bp, respectively. Annotation was performed manually based on the gene nomenclature of reference strains M129 (GenBank accession no. U00089.2) and 309.

The KCH-402 and KCH-405 genomes harbor characteristic variations in *orf6*, which encodes the cytadherence-related proteins P40 and P90 (9). Compared to the type 2 reference strain FH (GenBank accession no. CP010546.1), the variation in KCH-402 was in the P40 part of *orf6*, spanning an approximately 680-bp region. Meanwhile, the variation in KCH-405 was in a 35-bp region of the P90 part of *orf6*. These variations were probably generated by DNA recombination events between the *orf6* locus and RepMP5 repetitive sequences in the *M. pneumoniae* genome (10). Next, we inspected our *M. pneumoniae* strain collection and confirmed that three other type 2b strains carried *orf6* sequences identical to that of KCH-402. The KCH-405 *orf6* sequence was commonly found in type 2c strains but was also shared by several type 2a strains of our collection. A KCH-405-like *orf6* gene was also present in the draft genome sequence of the type 2d strain 3896 (GenBank accession no. LHPS00000000).

Comparative analysis of the KCH-402 and KCH-405 genomes with the reported* M. pneumoniae* genomes by whole-genome, single-nucleotide variation analysis revealed that the type 2b strain KCH-402 clustered within the type 2 strain clade and the type 2c strain KCH-405 was within a distinct clade comprising type 2a and 2c strains.

The polymorphism of both *orf6* and *p1* might be useful for strain classification and discrimination. The complete genome sequences of KCH-402 and KCH-405 may be used as reference sequences for type 2b and 2c strains and for detailed phylogenetic and epidemiological characterization of *M. pneumoniae* clinical strains.

### Accession number(s).

The complete genome sequences of strains KCH-402 and KCH-405 have been deposited in DDBJ/ENA/GenBank under the accession numbers AP017318 and AP017319, respectively.
